# A Feasibility Study on the Use of a Structured Light Depth-Camera for Three-Dimensional Body Measurements of Dairy Cows in Free-Stall Barns

**DOI:** 10.3390/s18020673

**Published:** 2018-02-24

**Authors:** Andrea Pezzuolo, Marcella Guarino, Luigi Sartori, Francesco Marinello

**Affiliations:** 1 Department of Agroforesty and Landscape, University of Padua, 35020 Legnaro, Italy; andrea.pezzuolo@unipd.it (A.P.); luigi.sartori@unipd.it (L.S.); 2 Department of Environmental Science and Policy, University of Milan, 20123 Milan, Italy; marcella.guarino@unimi.it

**Keywords:** dairy barn, body measurement, animal growth, low-cost sensor, 3-D sensor

## Abstract

Frequent checks on livestock’s body growth can help reducing problems related to cow infertility or other welfare implications, and recognizing health’s anomalies. In the last ten years, optical methods have been proposed to extract information on various parameters while avoiding direct contact with animals’ body, generally causes stress. This research aims to evaluate a new monitoring system, which is suitable to frequently check calves and cow’s growth through a three-dimensional analysis of their bodies’ portions. The innovative system is based on multiple acquisitions from a low cost Structured Light Depth-Camera (Microsoft Kinect™ v1). The metrological performance of the instrument is proved through an uncertainty analysis and a proper calibration procedure. The paper reports application of the depth camera for extraction of different body parameters. Expanded uncertainty ranging between 3 and 15 mm is reported in the case of ten repeated measurements. Coefficients of determination R² > 0.84 and deviations lower than 6% from manual measurements where in general detected in the case of head size, hips distance, withers to tail length, chest girth, hips, and withers height. Conversely, lower performances where recognized in the case of animal depth (R² = 0.74) and back slope (R² = 0.12).

## 1. Introduction

Frequent monitoring of animals’ body condition in quantitative terms is helpful in order to allow for early recognition of health anomalies and consequently decrease the amount of complications related to infertility, lameness, or other animal diseases [1,2,3,4]. With reference to young calves, the first months of life are critical, since animals’ growth may be affected by the appearance of several diseases or other stress factors, such as dehorning [5]. Similarly, in the case of young and adults’ cows, body condition measurement, and growth is important in order to monitor welfare state [6,7,8] and so to maintain high productivity levels [9,10].

Over the last fifty-years, the best way to measure individual animals body live weight, as well as their physical development, has been carried out through the use of traditional or electronic weighing systems [11,12]. However, such techniques are expensive in terms of time and involve considerable costs, which are borne by dairy farmers. As a result, visual inspections are often preferred and biometric analysis are made only in the case of visibly suffering animals, when preventive measures are not possible and curative interventions are less suitable and expensive [13,14].

An alternative approach, which allows for overcoming the limits associated with the use of manual direct measurements, is with the introduction of techniques based on optical detection instruments [15,16]. Literature reports several studies that propose implementation of vision technologies based on optical devices to automatically detect lameness [17], to evaluate dairy cows back posture [18], recognize abnormal behavior [19,20] and to measure body parameters [21,22,23,24]. 

In recent years, much research effort has been focused on the use of image analysis for automatic animal weighing or estimated body parameters combining one or more two-dimensional (2D) views to obtain the required measurements [25,26,27]. However, 2D images only offer two-dimensional projection of the animal and the lack of the third dimension in vision limits applications utilizing depth information [28]. Furthermore, two dimensional sensing is very much influenced by perspective, distance, and specific wavelength or applied filters [29], which can introduce relevant distortions in collected data, therefore the attention is nowadays focusing on three-dimensional reconstructions, obtained by two-dimensional sensors or on truly three-dimensional instruments. In the first case, stereoscopic techniques are typically adopted. Such approach relies on simultaneous collection of multiple 2D images taken from different perspectives, by means of CMOS or CCD cameras and three-dimensional (3D) models are reconstructed applying specific photogrammetry algorithms run by fast processors. Stereoscopic techniques have been successfully used to determine the three-dimensional models of pigs [30], rabbits [31], broiler chickens [32,33], and cattle [34]. One such successful example is a stereo vision system with six 2D cameras and three flash units used to capture the three-dimensional shapes of pigs [35]. However, this approach seeks to minimize instrumentation costs, but robust algorithms and accurate calibration procedures are needed.

The second case is based on different instruments based on different technologies, such as triangulation or time of flight, which allow direct reconstruction of 3D point clouds. The advantages associated to such approaches are typically related to high lateral and vertical resolution and ease of use, however relatively high investments are often required, while the harsh environment (e.g., temperature, dust) can reduce sensors life [36].

The present paper discusses metrological implementation of a low cost 3D depth camera technology to allow for extraction of quantitative cow body parameters, and specifically on hip and withers height, back slope, body length, hip distance, head size, and chest girth. 

Some works already mentioned application of the Kinect sensor in livestock applications. Maki reported quantification of body condition scoring after application of Kinect sensor on cows [37]; McPhee reported results on rump fat and muscle score from low and high muscled Angus cattle [38], while Kawasue took advantage of the 3D information depth to enhance the collection of thermal images from cow [39]. However, available literature is lacking with regard to the metrological performance: this is limiting very much actual application of the method, since it is not clear how much Kinect 3D data can support or replace manual measurements also with reference to different positions of animal body.

## 2. Material and Methods

### 2.1. Microsoft Kinect™ v1 RGB-Depth Camera

In the present work, low-cost depth-sensing cameras technology is applied. This is not a new invention, but recently, the usage of depth-sensing cameras technology has achieved an important diffusion, especially in the video-games productions. Therefore, this technology has spread quickly and high resolution depth cameras can be bought at a low-cost.

Specifically, a Microsoft Kinect™ v1 RGB-depth camera has been here implemented [40]. Since its launch in 2010, the sensor has been used in many applications, including agriculture [41,42] and livestock sector [43,44,45,46,47].

Microsoft Kinect™ captures synchronized colour and depth images at a rate of 30 frames per second (fps) using a RGB camera (8 bit VGA resolution with 640 × 480 pixel) aligned with a depth imager. The Kinect depth sensor is composed of an IR laser projector and a monochrome 640 × 480 pixel IR CMOS sensor [48].

A triangulation process permits to obtain a depth measurement [49]. The laser source emits a single beam, which is split into multiple beams by a diffraction grating to create a constant pattern of speckles projected onto the scene. This pattern is captured by the infrared camera and is correlated to a reference pattern. The reference pattern is obtained by capturing a plane at a known distance from the sensor, and is stored in the memory of the sensor. When a speckle is projected on an object whose distance to the sensor is smaller or larger than that of the reference plane, the position of the speckle in the infrared image will be shifted [50]. These shifts are measured for all of the speckles by a simple image correlation procedure, and then for each pixel the distance to the sensor can be retrieved from the corresponding disparity which allows reconstruction of the scene depth [51].

The Kinect sensor is priced less than 100 € and a new version allowing for a higher performance in terms of maximum permissible environmental light has been released in 2014, even though with a slight loss of resolution. Due to the recognized higher vertical resolution, Kinect V1 was preferred for the present work.

### 2.2. Sensors Positioning

Cattle, as well as cows’ body, have a complex three-dimensional shape, generally due to the incidence of hidden parts and overlapping body portions. Because of the cows’ frequent movements, the measurement is very difficult, in particular with regard to the head, legs, and tail of the animal.

**Figure 1:**
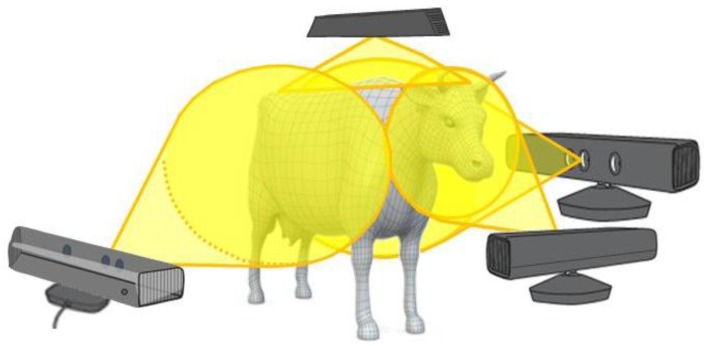
Perspective view of the animal, with relative positions of the three-dimensional (3D) optical sensors.

Therefore, data collection from multiple positions, with respect to the animals’ body, can contribute to a more complete and comprehensive analysis. As a result of an optimization phase, Figure 1 shows a reasonable compromise between the number of measurements and the completeness of the cows’ body cloud points. Four distinct scans positions are considered, respectively, to the side (one position on the left and one on the right sides), one on the top and the last in frontal of the animal.

It has to be noted that acquisition cannot be made simultaneously, because the model projected by one instrument could be interpreted in a wrong way by the detector of another sensor. However, this is not limiting since the Kinect may work with a frequency of over 10 Hz: therefore, if needed, opportunely positioning four Kinect instruments and synchronizing scan operations, a full measurement cycle could be in principle completed in less than 0.5 s. 

For the present research, which was aimed at defining the potential application of a low cost Structured Light Depth-Camera for frequent monitoring of calves and cows, the four sets of scans were obtained using one Kinect device fixed on a tripod. Tests were carried out in a barn, taking advantage of the posture featured by animals standing in front of the feeding alley. The Kinect instrument and the tripod were manually repositioned by the operator in the four reference positions, around the standing animal. The sensor was kept at an average distance ranging between 0.4 and 2 m from the animal (typically 1 m), with the sensor axis being roughly perpendicular to the captured animal portion. The measurement cycle took a time typically ranging between 10 and 15 seconds, during which the animal was minimally disturbed by the operator, which was moving at a minimum distance of about 1.5 m–3 m. As a consequence, the movements of the animal in such time interval were in general limited and negligibly affecting estimation of different body parameters. Indeed, the small relative movements of the animal and the lack of repeatability in sensor positioning and camera perspectives were in general not influencing results, thanks to the possibility of relocating collected point clouds, and compensating misalignments during the post processing phase [52].

### 2.3. Kinect Performance Verification

Microsoft Kinect™ is raising the interest of researchers involved in 3D reconstruction. However, it has been proven that the sensors output is not linear, and variability arises due to the specific instrument set up. For a quantitative application of the sensor, random and systematic errors have to be properly quantified and possibly compensated: therefore, specific calibration and performance analysis are necessary, eventually allowing for the definition of an uncertainty budget [53,54].

To this purpose, a substitution calibration approach was implemented, achieved through the measurement of a set of five reference polystyrene surfaces featuring hemispherical geometries, respectively with 50, 100, 150, 200, and 250 mm nominal radius [54]. A reference flat plane, with an average root mean square roughness lower than 0.3 mm and a deviation from flatness lower than 1 mm was additionally considered, in order to allow for estimation of background noise (BGN), quantified as the root mean square roughness of the vertical detected signal. 

The calibration procedure of Microsoft Kinect™ v1 was repeated at different working distances defined on the basis of actual animal measurement, in the range 400–2000 mm. Lateral resolutions ranging between 0.6 and 2 mm were found with a slight decrease of the performance with the distance. Similarly, the vertical resolution tends to decrease at higher distances with a 4 mm typical value at 2 m from the target surface. Background noise also increases due to the loss of vertical resolution, with BGN ranging between 1.1 and 5.5 mm. A summary of the main sensor performance parameters, averaged on 10 measurements repeated at three different distances is reported in Table 1. Correction of collected data was done after implementation of a correction model, as discussed in [55].

### 2.4. Measurement Uncertainty

The uncertainty budget was estimated both for the manual and non-contact analyses [56]. In the case of manual measurements, uncertainty is ascribable to the following sources: meter resolution and calibration, Abbe error (due to the misalignment between the meter scale and the cow body), definition of critical measuring points (e.g., hip positions, hooves to floor interface, etc.), and cow posture at the measuring time.

In the case of non-contact sensing, main uncertainty sources are again: sensor resolution and calibration, Abbe error (due to the installation slope of the sensor relatively to the animal surface), algorithm uncertainty on definition of reference points, and cows’ postures during the measuring session.

In order to allow for the estimation of uncertainty related to manual measurement, the manual meter underwent a calibration test on a reference standard (a metal rod with a calibrated 1 m length). Additionally, five different measurements were repeated by three different operators on three different animals (not included in the subsequent weight analysis) using both the manual meter and the Kinect sensor in order to estimate the reference points localization uncertainty contribution. Main results are reported in Table 2.

Combined standard uncertainties for different manual and Kinect measurements related to hip and withers height, back slope, hip distance, head size and chest girth have been computed considering uncorrelated input quantities, according to (1)

(1) $$U=k\cdot \sqrt{\underset{i}{\sum }{\left(t\cdot s_{i}\right)}^{2}}$$

where *s_i_* represent the different uncertainty contributions reported in Table 2, *t* is the conversion coefficient for standard deviation depending on specific distribution types, as reported in the last column in Table 2, and *k* = 1.645 defined on the basis of a 90% level of confidence. It is clear how in general multiple non-contact measurements feature lower expanded uncertainties (U = 3–15 mm) with respect to manual measurements (U = 7–40 mm), thus allowing for an increase of confidence and a reduction of uncertainty on measured parameters on average by 30–50%.

### 2.5. Data Acquisition

**Figure 2:**
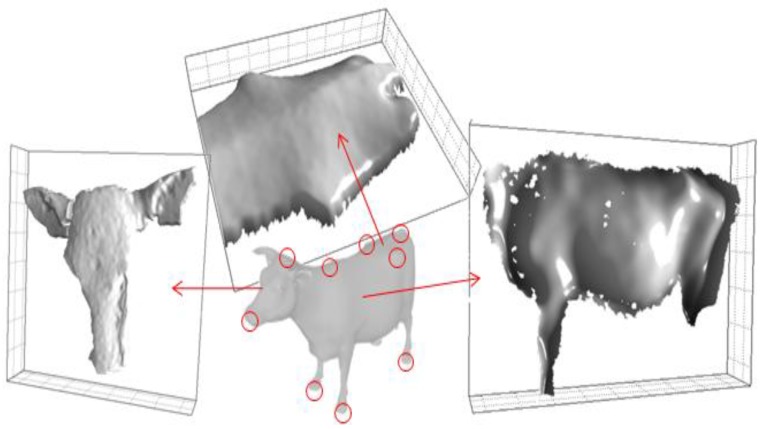
The system was implemented in order to allow for a fast extraction and monitoring of different body parameters, and in particular, hip and withers height, back slope, depth, hip distance, head size, and chest girth. Relevant points positions are indicated by red circles.

The system was implemented in order to allow a fast extraction and monitoring of different body parameters of 20 cattles (Table 3), and in particular hip and withers height, back slope, depth, hip distance, head size, and chest girth (Figure 2). Experiments were carried out in a free-stall barns with an average light intensity of 95 lx measured in the proximity of the animals using a luminance metre (Konica Minolta T10, Inc., Osaka, Japan).

For a comparison, the same cows underwent to a series of manual measures to evaluate the same body parameters. Specifically, a metric tape was used and data were collected positioning the tape in contact with or in close proximity of the relevant positions of the animal (hips, withers, head, etc.) and reading values by naked eye. Such an approach required a reduced distance (lower than 0.5 m) between the animal and the operator, causing an increase in the nervousness of cow. Chest girth was measured positioning the tape around the chest, immediately behind the front legs. In this case, it should be noted that out of four adult cows it has not been possible to perform the manual chest control, due to the frequent cow movements and to the danger of injuries for the operator that the measurement involved. Additionally, it should be noted that both in the case of young calves (age < 8 months) and adult cows (age > 8 months), the manual measurements are causing stress to the animals, therefore several attempts had to be repeated to get closer to the animal and collect a valid measurement (i.e., with the metric tape firmly positioned on the animal and readable numbers in the line of sight). 

On the other hand, in a few seconds, several tens of 3D data from the Microsoft Kinect™ v1 sensor could be collected. Specifically, 40 measurements were on average available for each animal and used for parameters extraction. 

### 2.6. Data Processing

**Figure 3:**
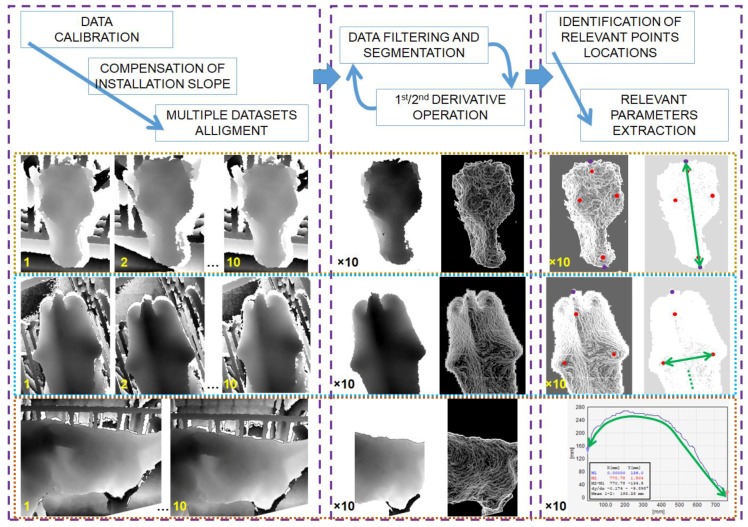
In the first row a flow chart representation of the applied methodology. In the bottom series of images three example of data extraction, respectively, for the head size, the hip distance, and chest girth, as indicated by green arrows.

3D data were post processed by means of commercially available software (SPIP™), undergoing three main blocks of operations, as depicted in Figure 3:

(i) data correction;
(ii) image filtering; and,
(iii) relevant parameters extraction.

In the first phase, a calibration was carried out in order to correct data sets and allow for subsequent extraction of quantitative values. Calibration was made taking advantage of calibration coefficients calculated through reference hemispherical geometries and applying a correction model [55]. A best fitting plane was also defined on the region of interest and used to calculate the average slope and allow rotation and easy relocation of the 3D data sets. In the second phase, flattened surfaces were segmented in order to isolate body portions and were subsequently processed through first and second derivative functions in order to produce gradient and curvature maps. In the third step, relevant positions were isolated applying thresholds or pass/non-pass filters. In particular, hips, withers, and tail head positions were identified as the local maximums (null gradients with negative curvatures); chest girth position was recognized as the thinnest body flank section (null gradient with positive curvature). Each body parameter was finally the average of values extracted from ten repeated 3D images captured on the same body portion.

The metrological quality of data extracted in the third step depends on the success of the first two steps. With regard to the calibration issue, details have already been given in previous paragraphs; the thresholding operation is probably the most challenging, sensibly affecting final data extraction. Thresholding operations were made on the basis of reference points, which were defined depending on the specific body portion. 

### 2.7. Body Parameters

In the case of hip, withers, and tail head, the reference position was achieved after a gradient operation, applied for automatic detection of the relative maximum, which was then applied in the calculation of hip distance, height, head size, body length, depth and back slope (Figure 2).

Reference hoof-floor interface was determined on the basis of the hoof highest slope variation, after application of a second derivative function. Such position was eventually applied for estimation of reference front height (at the withers level) and back height (at the hips level). Back slope was mathematically estimated as the arctangent of the rate between the difference between front and back height and the length of the animal from the hips to the withers. The depth was estimated as the minimum distance between the chest and the floor and calculated as the difference between the average height and the vertical projection of the girth. 

With reference to the head, since the sensor was positioned parallel to the cow head, Kinect maximum detection slope was usefully implement for the definition of the reference points. Since at the given working distance the applied sensor features a maximum detectable slope of 45°, higher angle positions are automatically set as void. Therefore, the border of the collected surface lies at the 45° slope position: reference points were consequently defined in the middle of such border, as the tangential point of a plane having a relative slope of 45° with respect to the cow face mean plane.

Data from different parameters arising from manual and non-contact measurements were compared by means of a linear regression. The aim of the analysis was not only to understand how much Kinect values can approximate manually captured ones, but also to highlight the most critical parameters. Analyses were carried out taking advantage of Microsoft Excel© statistical tools.

## 3. Results and Discussion 

**Figure 4:**
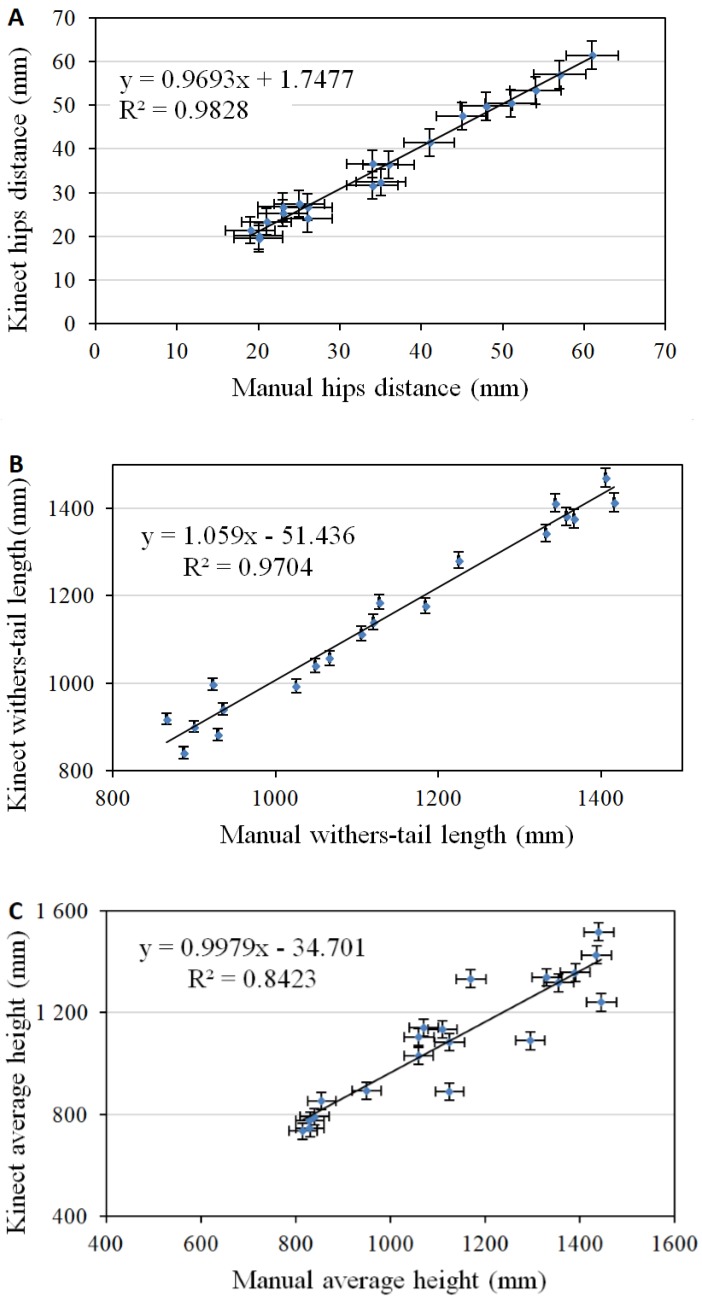

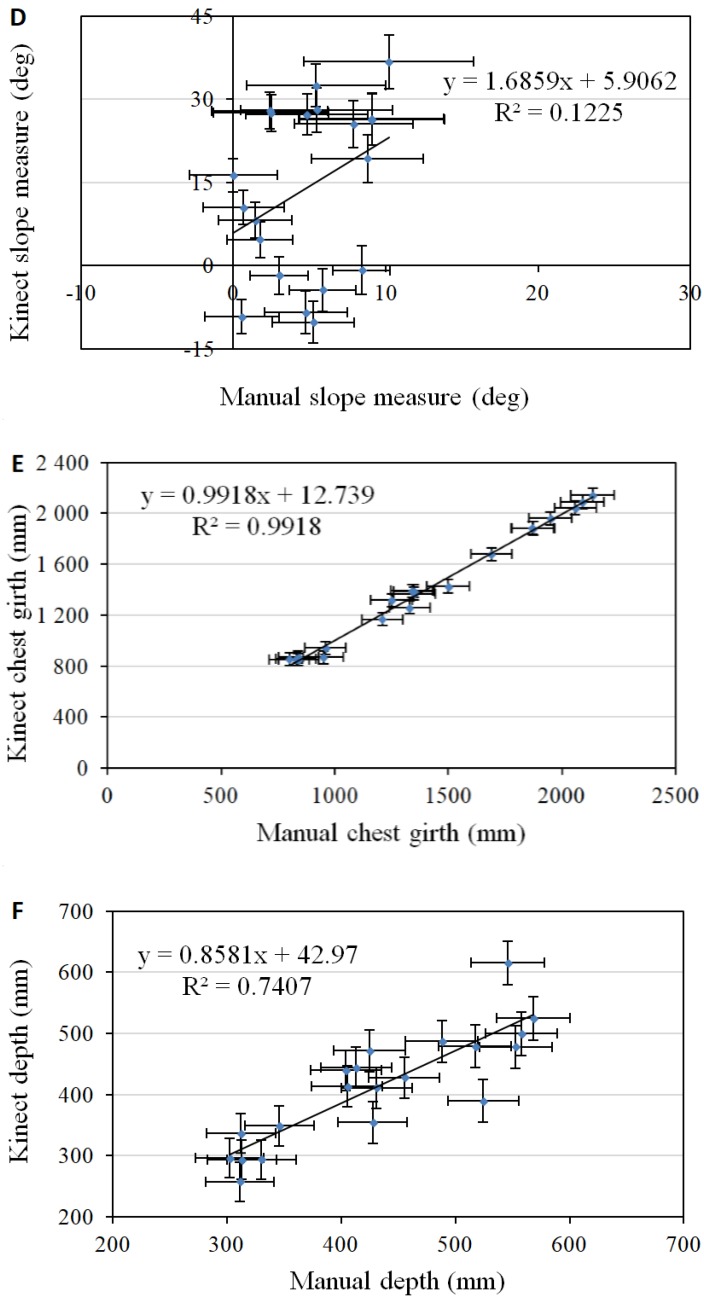

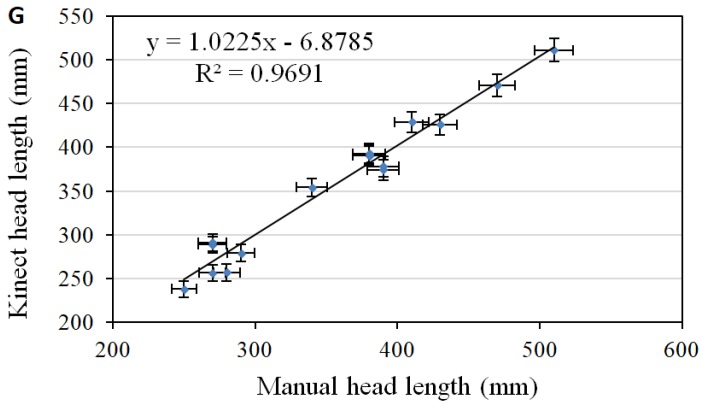
Assessment between different cow body parameters: (**A**) hip distance, (**B**) body length, (**C**) average height, (**D**) slope, (**E**) chest girth, (**F**) depth, and (**G**) head length. In the axis labels, “Manual” and “Kinect” refer, respectively, to manual and Kinect measurement.

The main results obtained from assessment between measurements carried out manually and measurement performed using 3D optical sensor are reported, respectively, in the graphs of Figure 4. Values are drawn with error bars whose dimension corresponds case by case with the expanded uncertainty calculated with Equation (1). Graphs report linear regression analysis, together with best fitting line equations and coefficients of determination to understand the relations between contact and non-contact data. 

In particular, with reference to the hip distance, results obtained by three-dimensional analysis are quite similar to the manual measurement, as shown by the linear regression coefficient close to one (m = 0.969), and the coefficient of determination R² = 0.983. As highlighted by the graph (Figure 4A), a good agreement can be predicted, especially for cows, where the hip bones are clearly more prominent. Conversely, in the case of young cattles, the deviations are larger but still within uncertainty limits, mainly due to the difficulties in the identification of exact hip positions: indeed, whenever the bone structures are not clearly visible, the localization of anatomical markers can be ambiguous, especially in the case of manual measurements. As Song [57], the consistency of locating these anatomical markers cannot be high due to the unclearness of the bone structure in young cattle.

A similar trend is highlighted in the body length analysis, estimated as the distance between withers and tail head length. In fact, an acceptable agreement with the manual method is shown by the linear regression coefficient equal to one and the coefficient of determination R² = 0.970 (Figure 4B).

Another parameter that is fundamental for determination and assessment, respectively, of growth and body weight is the height, in particular, in correspondence of the sides and the withers. To this respect, performed measurements have shown some limits; mainly due to a shadowing phenomenon on the ground caused by the cow body itself. As a consequence, in the case of images captured from the top of the animal downward, relatively large portions of the floor are not reconstructed, confusing height quantification. Also, it has to be pointed out that the Kinect sensor undergoes a non-linearity that introduces a distortion that is proportional to the measured length as reported in Table 2, and also discussed in some papers [58,59]: such non-linearity influences results, especially when performing measurements at large depths. The graph reported in Figure 4C shows how higher residuals cause a drop of the coefficient of determination down to R² = 0.842 for the average height. In order to improve such performance, it can be advisable to use lateral scan approaches, rather than measurements from above, thus taking advantage of the higher lateral measurement resolution and linearity, as compared to vertical ones.

Slope measurement (Figure 4D) is seldom used for body growth monitoring, however may represent an interesting indicator to monitor animals’ health problems, such as lameness or other hoof or legs disorders [60,61,62]. This parameter shows an inverse trend in terms of the coefficient of determination (R² = 0.122) than hip distance, body length, and height, due to uncertainty propagation. 

The chest girth can be measured using the combined measurements analysis made by top and lateral measurements. An acceptable agreement with the manual method, is shown by the linear regression coefficient close to one, and the coefficient of determination R² = 0.984 (Figure 4E). 

The depth parameter can also be determined by the difference between height value and chest projection. During the process of data validation, the determination coefficient was valued to be equal to R^2^ > 0.74, due to uncertainty propagation (Figure 4F).

Head measurement is less used for the body growth monitoring purposes, however it is easy accessible, therefore, this parameter should be taken into account in developments’ models. Head length was assessed both through manual analysis and 3D sensor. During the process of data validation, determination coefficient was valued to be equal to R^2^ > 0.96 (Figure 4G).

## 4. Conclusions

In the present paper, the Microsoft Kinect™ v1 sensor has been proposed, as a low cost instrument, to allow for body cow three-dimensional analysis, as well as non-invasive extraction of quantitative parameters. 

Hip distance, body length and height, chest girth, depth, back slope, and head size were evaluated on 20 cows’ and compared with the same measurements carried out manually. 

The experimental tests have shown relatively high coefficients of determination, in particular for parameters arising from three-dimensional measurements collected on low-depth surfaces, as is the case of hip distance, withers to tail head length or head size. Slightly lower accuracy is achieved when analyses are carried out on high aspect ratio body shapes or on purely three-dimensional shapes, as highlighted by average height or by chest girth data comparison. The lowest performances are observed when parameters values are estimated as a combination of multiple parameters, due to uncertainty propagation: this has been shown in the cases of back slope or depth experiments.

The method clearly needs pre-industrialization engineering activity to allow for automatic data collection and extraction. Additionally, studies are also needed in livestock environments to understand the effects of prolonged dust exposition on Kinect infrared projector or sensor, and the presence of high vibrations levels due for instance to the frequent passage of tractors or other vehicles in the proximity of the device installation. However, when considering that dairy farms are generally increasing their stock densities, the possibility of replacing manual with non-contact measurements is of great interest in order to allow for optimization of herd management based on individual cows’ health, welfare, and growth data monitoring, with no induced stress on animals.

## Figures and Tables

**Table 1:** Sensor scanning performance.

Distance (mm)	Lateral Range (mm)	Lateral Resolution (mm)	Vertical Resolution (mm)	BGN (mm)
400	415 × 310	0.65 × 0.65	0.7	1.1
1000	730 × 550	1.14 × 1.14	2	3.2
2000	1250 × 950	1.95 × 1.95	4	5.5

**Table 2:** Uncertainty sources.

Uncertainty Source	Uncertainty Estimation			
	Manual Meter (1 Measure)	Kinect Sensor (1 Measure)	Kinect Sensor (10 Measures)	Distribution
Lateral Resolution (mm)	1	0.65–2.0	0.3–0.7	Rectangular
Vertical Resolution (mm)	-	1.1–5.5	0.4–1.8	Rectangular
Background Noise (mm)	-	2.4	0.8	Triangular
Length Calibration Non-Linearity (%)	0.3	0.5	0.2	Normal
Abbe Error (%)	1.6	0.2	0.2	Normal
Reference Points Localization (mm)	4–15	10–18	2–6	Normal
Expanded Uncertainty U (mm)	7–30	16–40	3–15	Normal

**Table 3:** Dairy cattles information.

ID	Breed	Animal Category	Age (d)	Lactations Number
1	Holstein-Friesian	Calf	14	0
2	Holstein-Friesian	Calf	40	0
3	Holstein-Friesian	Calf	56	0
4	Holstein-Friesian	Calf	13	0
5	Holstein-Friesian	Calf	53	0
6	Holstein-Friesian	Calf	32	0
7	Red & white holstein	Calf	172	0
8	Red & white holstein	Calf	168	0
9	Holstein-Friesian	Calf	127	0
10	Holstein-Friesian	Calf	218	0
11	Holstein-Friesian	Calf	193	0
12	Holstein-Friesian	Cow	245	0
13	Holstein-Friesian	Cow	241	0
14	Holstein-Friesian	Cow	561	0
15	Holstein-Friesian	Cow	456	0
16	Holstein-Friesian	Cow	586	0
17	Holstein-Friesian	Cow	800	1
18	Holstein-Friesian	Cow	2224	3
19	Holstein-Friesian	Cow	1993	3
20	Holstein-Friesian	Cow	1386	2
